# No association between *FKBP5* gene methylation and acute and long-term cortisol output

**DOI:** 10.1038/s41398-020-0846-2

**Published:** 2020-06-02

**Authors:** Nina Alexander, Clemens Kirschbaum, Tobias Stalder, Markus Muehlhan, Susanne Vogel

**Affiliations:** 1 Department of Psychology, Faculty of Human Sciences, Medical School Hamburg, Hamburg, Germany; 2 Department of Psychology, Faculty of Science, Technische Universität Dresden, Dresden, Germany; 3 Clinical Psychology, University of Siegen, Siegen, Germany

**Keywords:** Psychology, Genetics

## Abstract

Prior studies identified DNA methylation (DNA_M_) changes in a regulatory region within the *FK506 binding protein 5* (*FKBP5*) gene as a crucial mediator of long-term negative health outcomes following early adversity. A critical mechanism underlying this link, in turn, has been suggested to be epigenetically induced dysregulation of the hypothalamic–pituitary–adrenal (HPA) axis. The purpose of this study was thus to investigate associations of *FKBP5* DNA_M_ with both acute and chronic cortisol output. Two hundred adults with differential exposure to childhood trauma (CT) were underwent a laboratory stressor (Trier Social Stress Test) and provided salivary samples for the analysis of acute cortisol stress responses. In addition, hair cortisol concentrations were determined as a valid measure of integrated long-term cortisol levels. Whole blood samples were drawn for DNA_M_ analyses of *FKBP5* intron 7 via bisulfite pyrosequencing. In contrast to most prior work, only healthy participants were included in order to disentangle the effects of trauma exposure per se from those related to mental disorders. First, our findings did not reveal strong evidence for a robust effect of CT on *FKBP5* intron 7 DNA_M_ status, even if genetic predisposition (rs1360780 genotype) was taken into account. Second, *FKBP5* DNA_M_ levels were found to be unrelated to acute cortisol stress reactivity and long-term cortisol concentration in hair. The failure to demonstrate a significant association between CT and *FKBP5* DNA_M_ in an exclusively healthy sample could be interpreted as suggesting that individuals’ mental health status may be a critical modulator of previously observed effects.

## Introduction

Early adversity has been repeatedly linked with the epigenetic state of genes that regulate major stress response systems, thereby promoting vulnerability to stress-related mental disorders^1^^,2^. One prominent example involves the *FK506 binding protein 5* (*FKBP5*) gene that acts as an important modulator of the hypothalamic–pituitary–adrenal (HPA) axis^3^. In response to stress exposure, rising cortisol levels rapidly induce *FKBP5* transcription via activating glucocorticoid-response-elements (GRE)^4,5^. The protein itself then provides an ultrashort negative feedback loop for glucocorticoid receptor (GR) signaling by reducing its cortisol binding affinity and impeding translocation of the receptor complex to the nucleus^6^. Consistent with these findings, genetic polymorphisms associated with increased *FKBP5* induction, most notably the rs1360780 T allele, induce GR resistance and impair negative feedback regulation of the HPA-axis^7^. This disruption in regulatory homeostasis ultimately results in chronically elevated glucocorticoid levels^3^. In turn, a long-term dysregulation of the HPA-axis (both in terms of hyper- and hypocortisolism) confers vulnerability for developing a wide range of stress-related mental disorders^8^. In line with this, the rs1360780 T allele (along with other high-induction FKBP5 alleles) has been identified as a risk factor for mental disorders in a recent candidate gene based meta-analysis^9^, in particular upon exposure to environmental adversity^7,10^. However, the latest genome-wide meta-analysis of depression could not replicate this finding^11^.

In search of potential molecular mechanisms, epigenetic modifications such as DNA methylation (DNA_M_) may underlie respective gene-environment (GxE) interactions^3,12^. In a landmark study, exposure to childhood trauma (CT) was found to be linked to allele-specific demethylation of a GRE located in intron 7 of the *FKBP5* gene, but only in those carrying the rs1360780 T allele^13^. Likewise, subsequent studies demonstrated comparably reduced *FKBP5* intron 7 DNA_M_ levels in cohorts exposed to different types of environmental adversity, including CT^13–16^, institutionalized care^17^, discrimination^18^, and cumulative life stress^19^, partly depending on rs1360780 genotype. However, others could not replicate this findings with regard to CT^20–22^ or have reported effects in the opposite direction, e.g. higher *FKBP5* intron 7 DNA_M_ levels in Holocaust survivors^14^.

Based on in vitro studies, it has been hypothesized that excessive glucocorticoid levels following stress exposure induce an active demethylation at the *FKBP5* intron 7 (particularly in risk allele carriers), thus enhancing the transcriptional response of FKBP5 to cortisol^13^. On a systemic level, this epigenetically induced upregulation of FKBP5 presumably leads to sustained GR resistance and a disruption of the HPA-axis feedback control, which may impose an increased risk to chronic hypercortisolism and related health outcomes^3^. However, data on associations of *FKBP5* intron 7 DNA_M_ state and functional HPA-axis activity in living humans is still sparse and largely relies on spot measurements of cortisol output, that are subject to large intraindividual variation. For example, studies applying single assessments of morning cortisol levels or cortisol awakening responses inconsistently report either negative^14^ or no correlations with *FKBP5* intron 7 DNA_M_^13,22^. However, the first study on long-term cortisol levels obtained evidence for a prolonged state of hypercortisolism related to *FKBP5* intron 7 demethylation based on awakening saliva cortisol levels averaged across 30+ days^23^.

The present study intended to replicate and extend prior research in several ways. First, we aimed to investigate the previously demonstrated interaction of CT by rs1360780 genotype on *FKBP5* intron 7 DNA_M._ As most prior studies included mixed samples of psychiatric patients (or samples that were not explicitly screened for confounding effects of psychopathology), our goal was to disentangle the effects of trauma exposure per se from those related to mental disorders^20,24^. For this, we recruited a sample of *n* = 200 healthy individuals with differential exposure to CT. Our second goal was to investigate associations of *FKBP5* intron 7 DNA_M_ with clinically relevant markers of both acute and chronic cortisol output that are characterized by substantial intraindividual stability. For this, participants were exposed to a standardized laboratory stressor and provided scalp hair for the analysis of hair cortisol concentrations (HCC), that have been proven a reliable and valid marker of long-term HPA-axis activity over a period of approximately three months^25^. Identifying long-term endocrine correlates of *FKBP5* DNA_M_ is a crucial step for advancing current understanding on how risk for stress-related disorders is conferred on a systemic level.

## Materials and methods

### Sample characteristics and procedure

Two hundred healthy participants of European descent (*n* = 100 females) aged 18–30 years were recruited by means of newspaper advertisements and flyers directed towards “healthy adults with a history of childhood trauma” and a control sample of “healthy adults without a history of childhood trauma”. In order to avoid a highly selective sample, we placed flyers at public places in both high and low socioeconomic status (SES) neighborhoods throughout Dresden, Germany, e.g. at bus stations, supermarkets, movie theaters and tanning salons. Part of this sample participated in a previously published study on genetic and epigenetic variation in the serotonin transporter gene and stress reactivity^26^. Exclusion criteria were current or past mental and/or physical disorders, medication intake (psychotropic drugs, substances known to influence HPA-axis activity, e.g. anti-inflammatory, allergy and thyroid medication), pregnancy, an irregular menstrual cycle and a body mass index (BMI) <17 or >30. Our rationale to include healthy participants only was to investigate associations of CT with *FKBP5* DNA_M_ profiles and acute/chronic cortisol output that are not confounded by unobserved variables that also determine an individual’s disease status. This is important given that psychopathology (as well as comorbidity, psychotherapy and drug intake) has been associated with marked changes in respective biomarkers and has not been controlled for in most prior studies on *FKBP5* DNA_M_^1,27^. During structured phone interviews, participants were screened for exclusion criteria (e.g., major health issues) and CT exposure by asking whether they had experienced any type of CT. All eligible individuals willing to participate were invited to an in-person screening interview that consisted of the diagnostic interview for psychiatric disorders—short version (Mini-DIPS) to assess point and lifetime prevalence of axis I disorders based on DSM IV^28^ and an in-house checklist on chronic physical diseases and medication to confirm eligibility. During this appointment, blood samples were drawn into EDTA tubes (Sarstedt, Nümbrecht, Germany) for DNA extraction and stored at −20 °C for no more than 6 months. The experimental stress induction was scheduled on a separate test day within close succession. The screening procedure continued until the planned sample of 100 exposed (50% females) and 100 non-exposed (50% females) individuals completed the study. For this, *n* = 622 individuals were screened via structured phone interviews and *n* = 211 individuals were invited to an in-person screening interview (where *n* = 11 participants were excluded due to physical and/or mental health issues).

The study was conducted in accordance with the Declaration of Helsinki and approved by the ethics committee of the Technische Universität Dresden. Participants provided written informed consent and received a monetary reward for participation.

### Standardized laboratory stress test

All participants underwent the Trier Social Stress Test (TSST), which is a standardized protocol to reliably elicit robust cortisol responses^29^. In short, the TSST consists of a public speaking (5 min) and a mental arithmetic task (5 min) performed in front of two evaluating panelists. During the experimental procedure, seven saliva samples were drawn including one baseline sample before onset of the TSST (after a 30 min resting period) as well as 1, 10, 20, 30, 45, and 60 min after stress induction. Experimental sessions started between 1330 and 1500 h to reduce the influence of diurnal cortisol variation. Participants were instructed to reschedule the session if they felt significantly impaired due to any reason and to refrain from physical exercising, smoking, eating, and drinking anything but water 1 h before test sessions. For females, the TSST was scheduled during the second half of the menstrual cycle only. To avoid creating a highly selective sample within this age group, smokers and oral contraceptive user were not excluded but these variables were treated as potential confounders.

### Salivary cortisol analysis

Saliva samples were collected by means of synthetic swabs (Salivettes, Sarstedt). Participants were instructed to chew on the swabs for 3 min to stimulate saliva flow. Synthetic rolls were transferred to plastic containers and stored at −20 °C. Saliva samples were thawed and centrifuged at 3000 r.p.m. for 3 min. Salivary-free cortisol levels were measured using commercially available chemiluminescence immunoassays (CLIA; IBL, Hamburg, Germany) with intra- and inter-assay precision of 3.0 and 4.2%, respectively.

### Hair cortisol analysis

Hair strands were cut as close as possible to the scalp from a posterior vertex position. HCC were analyzed from the 3 cm segment most proximal to the scalp to capture the cumulated cortisol secretion over the 3-month period prior to sampling. Washing procedure and extraction followed a previously published protocol^30^. Samples were analyzed by liquid chromatography coupled with tandem mass spectrometry. The LC-MS/MS system consisted of a Shimadzu LC-20AD high-pressure liquid chromatography unit, a Shimadzu SIL-20AC autosampler, and a Shimadzu CTO-20AC column temperature oven (Shimadzu Europa GmbH, Duisburg, Germany), which was coupled to an AB Sciex API 5000 turbo ion spray triple quadrupole tandem mass spectrometer (AB Sciex Germany GmbH, Darmstadt, Germany). The system was controlled by AB Sciex Analyst software (version 1.5.1). Intra-assay and inter-assay coefficients of variance were between 3.7% and 8.8%, respectively. Three individuals were excluded from further HCC analysis due to insufficient hair length (*n* = 2) and outliers (±3 SD from the mean, *n* = 1).

### Assessment of childhood trauma

CT was assessed by the Short Form of the Childhood Trauma Questionnaire (CTQ), a widely used retrospective measure of CT with high internal consistency, reliability and criterion validity in clinical and community samples^31^. The CTQ manual provides cut-off scores for none, low-moderate, and moderate-severe exposures to five trauma categories including emotional abuse (EA), physical abuse (PA), sexual abuse (SA), emotional neglect (EN), and physical neglect (PN)^31^. Based on these cut-off scores, composite scores were calculated across the five trauma categories to group participants into those with no (subsequently referred to as controls), mild-moderate (liberal cut-off scores: EA: > 9, PA: > 8, SA: > 6, EN: > 10, and PN: > 8) and moderate-severe (conservative cut-off scores: EA: > 13, PA: > 10, SA: > 8, EN: > 15, and PN: > 10) exposure to at least one type of CT.

### Bisulfite pyrosequencing

**Fig. 1:**
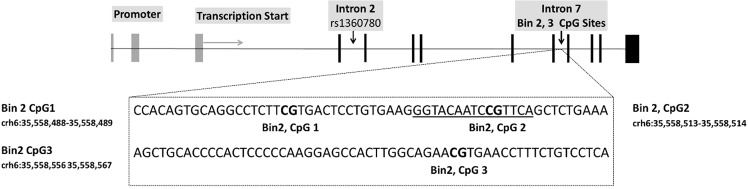
Schematic representation of the human *FKBP5* gene. The upper panel depicts the *FKBP5* locus in 5′–3′ orientation. Black bars indicate the 11 exons. The lower panel shows the targeted region analyzed by bisulfite pyrosequencing (intron 7 bin 2, comprising 3 CpG sites marked in bold letters). The functional consensus glucocorticoid response element (GRE) is underlined.

Quantitative DNA_M_ analysis was performed by Varionostic GmbH (Ulm, Germany). The targeted region contained three CpGs sites corresponding to *FKBP5* intron 7 bin 2^13^ that are located within, or in proximity to, a functional consensus GRE (Fig. 1). After extensive screening of functionally relevant regions within the *FKBP5* gene, DNA_M_ levels at these particular sites were previously found to be sensitive to childhood trauma^13^. A detailed description of the bisulfite pyrosequencing protocol with amplicon and sequencing primers has been published elsewhere^13^. In brief, genomic DNA extracted from EDTA whole blood was bisulfite-treated using the EZ DNA Methylation Gold Kit (Zymo Research, Range, CA, USA). Subsequent pyrosequencing was performed on the Q24/ID System including three human methylation standards (0%, 50%, 100%). Percent DNA_M_ at each CpG site was quantified using the PyroMark Q24 software (Qiagen) with standard quality-control settings implemented in the software. 199 samples passed quality control for all CpG sites investigated. For subsequent analyses, mean percent DNA_M_ levels across the three CpG sites analyzed within *FKBP5* intron 7 bin 2 were calculated. Mean *FKBP5* DNA_M_ levels were 83.45 (SD: 3.87, CpG site 1: 97.74 ± 3.08, CpG site 2: 93.31 ± 9.41, and CpG site 3: 59.30 ± 5.46). Kolmogorov–Smirnov tests showed that DNA_M_ data did not follow a normal distribution (all *p*’s < 0.001), even after logit-transformation (all *p*’s < 0.001). In line with previous publications and due to the relatively large sample, we nonetheless used parametric statistical methods, but verified that all results hold with non-parametric tests, if appropriate alternatives were available.

### *FKBP5* rs1360780 genotyping

Genotype data were available from a subset of *n* = 175 individuals. Participants were genotyped by means of MALDI-TOF mass spectrometry using the MassARRAY-4 system, Complete iPLEX Gold Genotyping Reagent Set and the GenoTYPER software (Agena Bioscience). For reasons of quality control, 40% of the samples were additionally genotyped by Real-Time-PCR using a LightCycler 480 System (Roche Diagnostics, Mannheim, Germany). Primers and hybridization probes were customized produced (TIB MOLBIOL, Berlin, Germany). Respective Real-Time PCR results 100% replicated the MASS-Array findings. Genotype frequencies (TT = 17, CT = 60, and CC = 98) were in Hardy–Weinberg equilibrium (*p* > 0.05).

### Statistical analyses

Statistical analyses were conducted using SPSS (Version 25.0. IBM, Chicago, IL, USA). All statistical tests were two-tailed with alpha set at *p* < 0.05. Potential confounders with regard to CT, *FKBP5* DNA_M_, cortisol stress responses, and HCC were identified by means of analysis of variance (ANOVA), Chi-Square (*χ*^2^) test or Pearson correlations. Associations between CT, rs1360780 genotype and their interaction on mean and site-specific *FKBP5* DNA_M_ levels were assessed by ANOVAs. Regarding CT, we started statistical analyses using a conservative CT cut-off score for group comparison (comparing participants with vs. without moderate-severe exposure) and subsequently tested whether results generalize to milder forms of CT trauma using a more liberal cut-off score (comparing participants with at least mild-moderate vs. no exposure). In addition, a dimensional CTQ abuse score was applied to explore (risk-allele specific) correlations between different types and overall CT with mean and site-specific *FKBP5* DNA_M_ levels. Next, covariate adjusted regression models were set up to test whether *FKBP5* DNA_M_ is related to differences in cortisol stress reactivity (indexed by the cortisol curve with respect to the ground (AUC_G_) according to Pruessner and coworkers^32^) and HCC levels.

## Results

### Study sample

Supporting our selective recruitment, our sample consisted of 105 individuals exposed to CT and 95 individuals without CT. Of those participants reporting CT exposure, 73 individuals experienced mild-moderate CT exposure, and 32 individuals reported moderate-severe exposure. The most commonly reported form of CT was EN (while SA was least common), and approximately half of the traumatized participants (*n* = 50) reported multiple trauma types. The different trauma groups did not differ in sex distribution, age, years in school, body mass index, or use of oral contraceptives (Table 1). However, participants with moderate-severe CT smoked more frequently than the other groups.

**Table 1:** Sample characteristics of participants exposed to none, mild-moderate, or moderate-severe childhood trauma.

	Childhood trauma (CT) exposure			
	None (*n* = 95)	Mild-moderate (*n* = 73)	Moderate-severe (*n* = 32)	*p* value
Sex (% female)	52.6	43.8	56.3	0.396
Age (mean, SD)	23.25 ± 2.82	24.27 ± 2.77	23.81 ± 3.01	0.069
Education (%)^a^				0.105
<10 years in school	5.3	1.4	3.1	
=10 years in school	28.4	19.2	28.1	
>10 years in school	66.3	79.5	68.8	
BMI (mean, SD)	22.46 ± 2.15	22.39 ± 2.26	22.05 ± 2.34	0.660
Smoking (% yes)	32.6	26.0	53.1	0.024
Oral contraception (% of female sample)	58.0	53.1	33.3	0.201
rs1360780 (% T carriers)^b^	43.5	41.8	52.2	0.686
CTQ sum score	27.76 ± 2.23	33.89 ± 3.53	48.87 ± 10.20	<0.001
Emotional abuse	5.81 ± 1.01	7.08 ± 2.01	11.91 ± 4.47	<0.001
Physical abuse	5.16 ± 0.32	5.22 ± 0.63	7.16 ± 2.81	<0.001
Sexual abuse	5.00 ± 0.00	5.21 ± 0.55	6.00 ± 2.41	<0.001
Emotional neglect	6.62 ± 1.42	9.75 ± 2.41	14.25 ± 3.17	<0.001
Physical neglect	5.21 ± 0.48	6.63 ± 1.40	9.56 ± 2.05	<0.001

*BMI* body mass index, *CTQ* childhood trauma questionnaire.

^a^Due to the low frequencies of <10 years of school, group comparisons are based on ≤10 years vs. >10 years in school.

^b^Genotyping data was available for *n* = 175 individuals.

### No effects of childhood trauma, rs1360780 genotype and their interaction on *FKBP5* DNA_M_

We first identified potential covariates (see Table 1) that were associated with *FKBP5* DNA_M_. Age, sex, years in school, BMI, oral contraceptive use, and smoking were all unrelated to differences in mean *FKBP5* DNA_M_ (all *p*’s > 0.20) and were thus not included as covariates in subsequent analyses.

**Fig. 2:**
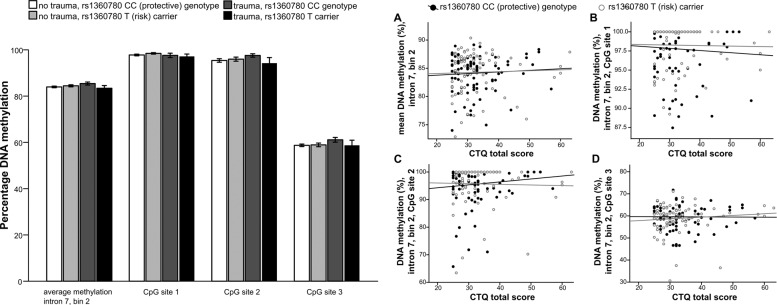
Effects of childhood trauma, rs1360780 genotype and their interaction on FKBP5 methylation. Left part: mean and site-specific DNA methylation levels in *FKBP5* intron 7, bin 2 grouped by rs1360780 T allele carrier status and moderate-severe childhood trauma exposure according to the childhood trauma questionnaire (CTQ). Data show mean±s.e.m. Right part: correlation between DNA methylation levels in *FKBP5* intron 7 bin 2 and total CTQ scores in *FKBP5* rs1360780 T (risk) allele carriers and protective genotype (CC) carriers. A mean methylation at *FKBP5* intron 7, bin 2; B methylation at CpG site 1; C methylation at CpG site 2; and D methylation at CpG site 3.

Next, we tested whether *FKBP5* DNA_M_ was affected by CT, rs1360780 genotype, and their interaction. In contrast to previous findings, we found no significant main effect of moderate-severe CT (*F*_(1,170)_ = 0.071, *p* = 0.790, Fig. 2, left part) or rs1360780 T allele carrier status (*F*_(1,170)_ = 1.033, *p* = 0.311, Fig. 2, left part) on average *FKBP5* DNA_M_. Similarly, the CT × rs1360780 T allele status interaction did not reach significance (*F*_(1,170)_ = 2.719, *p* = 0.101, Fig. 2, left part). Additionally, we ran the same analysis separately for each CpG site. Again, we found no significant effect of moderate-severe CT, rs1360780 T allele status, or their interaction on DNA_M_ at CpG site 1 (all *p* > 0.20, Fig. 2, right part), CpG site 2 (all *p* > 0.20, Fig. 2, right part), or CpG site 3 (all *p* > 0.30, Fig. 2, right part). Similar results were obtained when DNA_M_ analyses were conducted based on logit-transformed values (data not shown).

We were then interested in whether mild exposure to CT would affect *FKBP5* DNA_M_ as it has been shown in some prior studies^15^. To this end, we analyzed the effect of having experienced at least one mild CT (vs. none) and rs1360780 T allele status on *FKBP5* DNA_M_ (group sizes: carrier without CT = 37, with CT = 40; non-carrier without CT = 47, with CT = 50). Again, we found no effect of CT (*F*_(1,170)_, *p* = 0.808), rs1360780 T allele status (*F*_(1,170)_, *p* = 0.750), or their interaction (*F*_(1,170)_, *p* = 0.431) on *FKBP5* DNA_M_ (data not shown). Likewise, we found no effect of at least mild CT exposure, rs1360780 T allele status, or their interaction on DNA_M_ at the specific CpG sites (site 1: all *p* > 0.20, site 2: all *p* > 0.40, site 3: all *p* > 0.60). Similar results were obtained when DNA_M_ analyses were conducted based on logit-transformed values (data not shown).

Next, we also tested for a (risk allele-specific) correlation between total CT exposure and *FKBP5* DNA_M_. Using a dimensional CTQ summary score, we observed no significant correlation between CT and average *FKBP5* DNA_M_ (*r* = 0.111, *p* = 0.118, Supplementary Table 1). When conducting exploratory analyses for specific CpG sites and all types of trauma (Supplementary Table 1), we observed a nominally significant negative association between DNA_M_ at CpG 2 and CTQ summary score (*r* = −0.171, *p* = 0.016), physical abuse (*r* = −0.168, *p* = 0.018) and emotional abuse (*r* = −0.172, *p* = 0.015). However, none of these associations remained significant when applying a Bonferroni correction for three CpG sites × five CTQ dimensions with an alpha level of 0.05/15 (*p* < 0.0034) or when we re-ran the analyses with non-parametric tests (Spearman’s correlation).

When taking rs1360780 genotype into account, we further found no significant correlation between CTQ summary score and average *FKBP5* DNA_M_ and in either genotype group (risk allele (T) carriers: *r* = 0.036, *p* = 0.753, *n* = 77; protective genotype (CC): *r* = 0.053, *p* = 0.539, *n* = 97, Fig. 2, right part, Supplementary Tables 2 and 3) and no significant difference between these correlations (Fisher *z* = 0.17, *p* = 0.865). Finally, we also ran these dimensional analyses separately for the individual CpG sites in intron 7 bin 2, but again found no significant association with total CT exposure (all *p*’s > 0.20, Fig. 2, right part, Supplementary Tables 2 and 3). Regarding effects of specific type of trauma, the only significant result indicates a negative association of sexual abuse and DNA_M_ at CpG site 3 in T allele carriers (*r* = −0.34; *p* = 0.0025, significant after Bonferroni correction, Supplementary Table 2). Again, this association did not hold when we re-ran the analyses using a non-parametric Spearman’s correlation (*r* = −0.151, *p* = 0.188).

### No association of *FKBP5* methylation with acute and chronic cortisol output

Next, we investigated whether *FKBP5* DNA_M_ would be associated with an acute (i.e., cortisol stress response to the TSST) and chronic (i.e., HCC) state of hypercortisolism. First, we verified that the TSST successfully increased cortisol levels in our sample (main effect of time: *F*_(2.05, 407.74)_ = 198.95, *p* < 0.001, *η*_p_^2^ = 0.50). As previously reported^26^, we observed no main effect of moderate-severe CT on cortisol stress reactivity (CT: *F*_1,195_ = 0.465, *p* = 0.496; CT × time: *F*_6,2.26_ = 0.737, *p* = 0.495; Supplementary Fig. 1). Dimensional analysis revealed a nominally significant negative correlation between cortisol stress reactivity (AUC_G_) and sexual abuse (*r* = −141, *p* = 0.048, uncorrected), but not with other types of trauma or CTQ sum score (all *p*’s > 0.612, Supplementary Table 4).

**Fig. 3:**
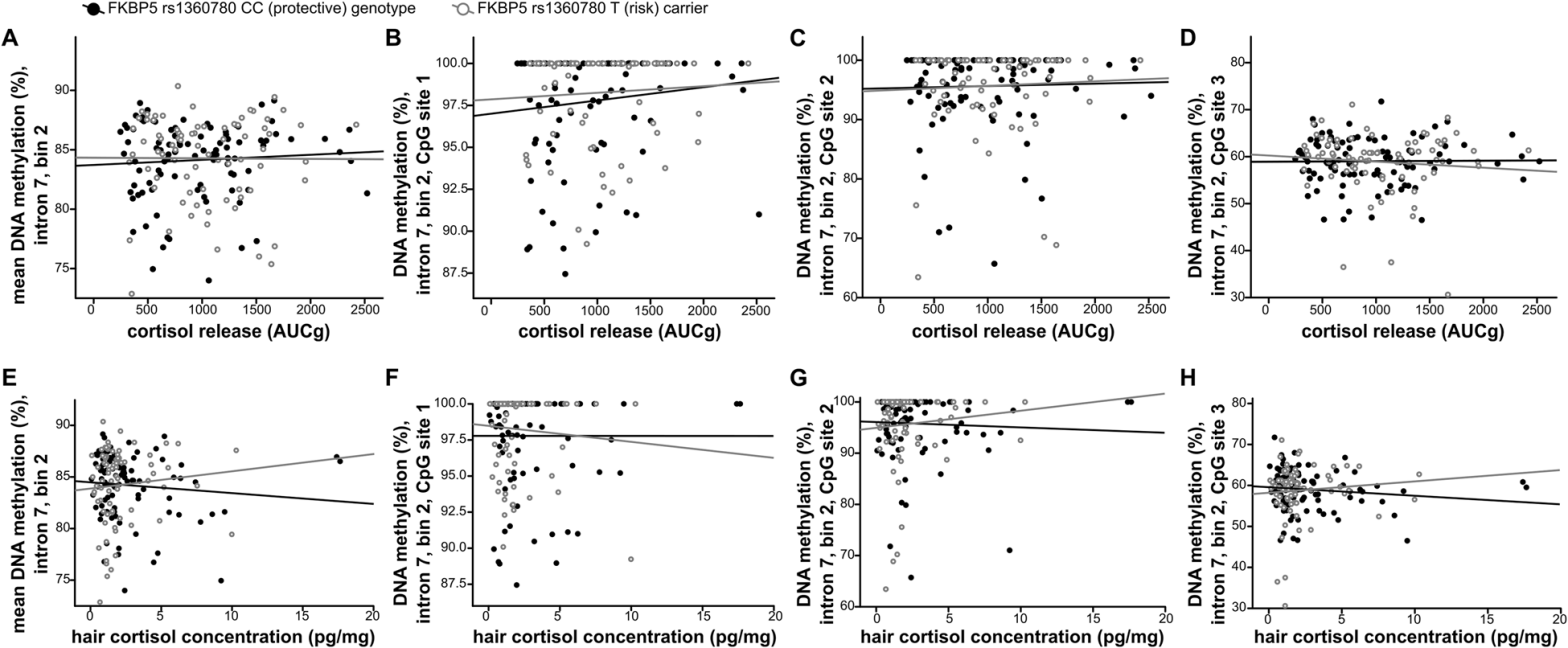
Association of FKBP5 methylation with acute and chronic cortisol output. Upper part: correlation between DNA methylation in *FKBP5* intron 7 bin 2 and cortisol release in the Trier Social Stress Test (area under the curve with respect to the ground, AUC_G_) in *FKBP5* rs1360780 risk allele carriers and protective genotype carriers. **a** Mean methylation in *FKBP5* intron 7 bin 2; **b***FKBP5* methylation in CpG site 1; **c***FKBP5* methylation in CpG site 2; **d***FKBP5* methylation in CpG site 3. Lower part: correlation between DNA methylation in *FKBP5* intron 7 bin 2 and hair cortisol concentration in *FKBP5* rs1360780 risk allele carriers and protective genotype carriers. **e** mean *FKBP5* methylation in intron 7 bin 2; **f***FKBP5* methylation in CpG site 1; **g***FKBP5* methylation in CpG site 2; **h***FKBP5* methylation in CpG site 3.

We then set up a regression model to test whether mean *FKBP5* DNA_M_ is related to differences in AUC_G_ cortisol stress reactivity. Similar to previous studies, we also included oral contraceptive use, smoking status, and baseline cortisol levels, as they showed at least trend-level significant associations with the cortisol AUC_G_ (oral contraceptive use: *p* = 0.017, *R*^2^_corr_ = 0.081; smoking status: *p* = 0.053, *R*^2^_corr_ = 0.015; baseline cortisol levels: *p* < 0.001, *R*^2^_corr_ = 0.180). In contrast, age, sex, and BMI were not significantly related to cortisol AUC_G_ and were thus not included in the regression analysis (all *p*’s > 0.15). The resulting model was significant in predicting cortisol stress reactivity (*F*_(4,194)_= 8.33, *p* < 0.001, *R*^2^_corr_ = 0.259). However, although oral contraceptive use (*p* < 0.001), smoking status (*p* = 0.026), and baseline cortisol levels (*p* < 0.001) were predictive of cortisol release under stress, mean *FKBP5* DNA_M_ was not (*p* = 0.455, Table 2a, Fig. 3, upper part). Again, we also tested if DNA_M_ at specific CpG sites was associated with cortisol release by replacing mean *FKBP5* DNA_M_ by DNA_M_ at the three individual CpG sites in the regression model. However, the results were largely comparable and site-specific DNA_M_ did not significantly predict cortisol stress reactivity (all *p* ≥ 0.173, Fig. 3, upper part), irrespective of rs1360780 genotype. Likewise, results remained stable when DNA_M_ analyses were conducted based on logit-transformed values (data not shown).

**Table 2:** Linear regression models investigating the effects of *FKBP5* DNA methylation on [A] acute and [B] chronic cortisol release.

A: Dependent variable: cortisol stress response to the Trier Social Stress Test (AUC_G_).			
	*β*	*T*	*p*
Model 1: average *FKBP5* DNA methylation			
(Constant)	–	1.897	0.059
* FKBP5* intron 7 bin 2 methylation	−0.046	−0.748	0.455
Oral contraceptive use	−0.258	−4.179	<0.001
Smoking status	−0.137	−2.239	0.026
Baseline cortisol levels	0.408	6.637	<0.001
Model 2: site-specific *FKBP5* DNA methylation			
(Constant)	–	0.386	0.700
* FKBP5* CpG site 1 methylation	0.056	0.796	0.427
* FKBP5* CpG site 2 methylation	−0.095	−1.366	0.173
* FKBP5* CpG site 3 methylation	−0.007	−0.105	0.916
Oral contraceptive use	−0.258	−4.057	<0.001
Smoking status	−0.143	−2.298	0.023
Baseline cortisol levels	0.400	6.443	<0.001

B: Dependent variable: hair cortisol concentration (HCC)			
Model 3: average *FKBP5* DNA methylation			
(Constant)	–	0.198	0.843
* FKBP5* intron 7 bin 2 methylation	0.041	0.559	0.577
Age	−0.004	−0.049	0.961
Sex	−0.159	−1.547	0.124
Oral contraceptive use	−0.022	−0.242	0.809
Hair treatment	0.010	0.103	0.918
Hair washing frequency	−0.040	−0.522	0.602
Model 4: single CpG site *FKBP5* DNA methylation			
(Constant)	–	0.753	0.453
* FKBP5* CpG site 1 methylation	−0.050	−0.612	0.541
* FKBP5* CpG site 2 methylation	0.088	1.048	0.296
* FKBP5* CpG site 3 methylation	0.004	0.050	0.960
Age	0.000	0.007	0.995
Sex	−0.140	−1.317	0.189
Oral contraceptive use	−0.029	−0.314	0.754
Hair treatment	0.006	0.062	0.950
Hair washing frequency	−0.035	−0.449	0.654

With regard to long-term cortisol output, initial analysis revealed no main effect of moderate-severe CT on HCC (*F*_1,189_ = 0.126, *p* = 0.723). Likewise, dimensional analyses revealed no significant correlation between HCC und CTQ sum score as well as specific types of trauma (all *p*’s ≥ 0.390, Supplementary Table 4). To finally test whether *FKBP5* DNA_M_ predicts long-term cumulative cortisol release as assessed by HCC, we set up a regression model including mean *FKBP5* DNA_M_ and relevant predictors for HCC as identified in a recent meta-analysis^25^. Importantly, mean *FKBP5* DNA_M_ at intron 7 bin 2 did not predict HCC (*p* = 0.577, Table 2b, Fig. 3, lower part). Finally, we again tested whether DNA_M_ at specific CpG sites is associated with HCC. Thus, we set up a similar model but replaced mean *FKBP5* DNA_M_ by DNA_M_ at the three CpG sites investigated. However, site-specific *FKBP5* DNA_M_ also did not significantly predict HCC (all *p* ≥ 0.296, Table 2B, Fig. 3, lower part).

## Discussion

Motivated by prior landmark studies, we first aimed to replicate previously observed GxE interactions on epigenetic changes within a regulatory region of the *FKBP5* gene in a sample of healthy adults with differential exposure to CT. Second, we were the first to use both experimental stress induction and hair steroid analysis to further elucidate the proposed dysregulation of the HPA-axis related to epigenetic changes in *FKBP5*. In summary, the current study failed to provide robust evidence for a general or site-specific effect of either CT, rs1360780 genotype or their interaction on *FKBP5* intron 7 DNA_M_. Although we observed nominally significant correlations between dimensional CTQ summary, physical abuse, as well as emotional abuse scores and DNA_M_ at one specific CpG site (CpG4) in the expected direction, none of these associations remained significant after correcting for multiple testing. Interestingly, we observed a negative association of sexual abuse and DNA_M_ at CpG site 3 in rs1360780 T allele carriers only. However, this finding should be interpreted with extreme caution, given that (1) sexual abuse scores were overall extremely low in the current sample and (2) given that DNA_M_ data were heavily skewed and the association did not hold when we re-ran the analyses using a non-parametric test. The second major finding of our study indicates that *FKBP5* DNA_M_ levels were unrelated to acute cortisol stress reactivity and long-term cortisol concentration in hair.

These findings seemingly contradict several studies demonstrating that adverse environmental exposures relate to long-lasting robust alterations of *FKBP5* DNA_M._ Klengel and colleagues were the first to demonstrate a significant demethylation of *FKBP5* intron 7 in adult trauma survivors compared to unexposed controls, particularly in those carrying the rs1360780 T allele^13^. Likewise, lower *FKBP5* intron 7 DNA_M_ has been linked to a history of CT^15,16^ and the time spent in institutional care^17^, albeit independent of rs1360780 genotype. Later research then suggested that *FKBP5* DNA_M_ is also sensitive to other types of stress exposure such as adult discrimination^18^ and cumulative life stress during childhood^19^. In contrast, other studies failed to replicate robust associations of CT and *FKBP5* DNA_M_ intron 7 DNA_M_^21^, among them the largest study to date comprising 3965 individuals^20^. Notably, the sample size of the current study is comparable or even exceeds those of prior studies that reported significant associations of CT (with *n*’s ranging from 76 to 174, respectively) and had 82% power to detect medium effect sizes according to a post-hoc power calculation using G*Power^33^. Importantly, however, a critical difference to our study is that respective samples were not explicitly screened for potential confounding effects of psychopathology which makes it hard to disentangle direct effects of CT from those related to psychiatric conditions. Indeed, epigenetic changes in general^1^ and a hypomethylated *FKBP5* intron 7 in specific^20,24^ have been repeatedly linked to stress-related psychopathological symptoms. In the light of these findings, the failure to replicate a robust association between CT and *FKBP5* DNA_M_ in an exclusively healthy, well-educated sample could be interpreted as suggesting that individuals’ disease status may be a critical modulator of previously observed effects. Another possible reason for non-replication relates to differences in the severity of CT, as in contrast to our study, most prior work relied on heavily traumatized populations with low SES^13,16^. As such, contextual stressors related to low SES might be crucial for *FKBP5* DNA_M_ changes to establish in the aftermath of CT, as recently suggested^19^. Moreover, our sample differed from those of some previous studies regarding ethnicity and the prevalence of specific types of CT (e.g., sexual abuse was highly prevalent in the initial study by Klengel et al.^13^) which might further contribute to conflicting findings. Alternatively, the possibility that publication bias has an effect on findings in this field cannot be ruled out and should be evaluated in future meta-analysis.

Although we were unable to identify a robust link with CT, interindividual variation in *FKBP5* DNA_M_ in our sample was comparable to those previously observed, which provides a solid prerequisite to evaluate an associated dysregulation of the HPA-axis. In first ex vivo experiments by Klengel and colleagues^12^, *FKBP5* intron 7 demethylation was found to enhance the ultra-short feedback loop between the GR and FKPB5, thereby promoting GR resistance. This, in turn, has been proposed to result in an epigenetically induced state of hypercortisolism that may convey an increased vulnerability to stress-related disorders. Alternatively, a preexisting state of hypercortisolism may also be the cause of respective epigenetic changes as a demethylation of *FKBP5* intron 7 was also shown in human hippocampal progenitor cell lines following dexamethasone treatment^13^. Likewise, comparably lower levels of *FKBP5* DNA_M_ were reported in patients suffering from Cushing’s disease, that is characterized by a tumor-induced hypercortisolism^34^. First studies applying spot measurements to investigate associations of *FKBP5* DNA_M_ status and cortisol output in living humans, however, yielded inconsistent result. While two studies reported no associations of *FKBP5* intron 7 DNA_M_ with morning cortisol levels^13^ and cortisol awakening responses^22^, only one study observed a negative correlation with a single measure of wake-up (but not bedtime) salivary cortisol^14^. One important limitation of single/limited cortisol measures refers to their inability to derive information on long-term HPA-axis dysregulation as cortisol secretion underlies substantial state/situational fluctuations^25^. To address this gap, the current study uses a combination of clinically relevant dynamic (cortisol responses to experimental stress) and chronic (HCC) cortisol markers that are both characterized by significant intraindividual stability^35,36^. Contrary to our a priori hypothesis, were unable to detect any relations of *FKBP5* DNA_M_ with acute and chronic cortisol output. This finding stands in contrast with the only study published so far that investigated long-term salivary cortisol changes related to *FKBP5* DNA_M_^22^. More precisely, this study reports a negative correlation of *FKBP5* DNA_M_ at a specific site of *FKBP5* intron 7 (corresponding to CpG 1 in the current study) with averaged cortisol awakening levels sampled over a month. Again, the failure to replicate an association of DNA_M_ and cortisol output is unlikely to result from insufficient power given that our sample size yielded a power of 99% to detect medium-sized effects and exceeds those of previous studies (where *n*’s ranged from 22 to 75, respectively).

Several limitations of the present study should be acknowledged. First, our findings are based on retrospective self-report measures of CT, which could be subject to bias. Second, peripheral measures of *FKBP5* DNA_M_ may not necessarily generalize to neural tissue. However, post-mortem^37^ and studies on living humans^38^ demonstrated significant correlations of DNA_M_ profiles in peripheral and neural cells, providing a solid base for analyzing DNA_M_ in blood. In addition, rodent models revealed that corticosterone exposure induced comparable changes of *FKBP5* DNA_M_ profiles in peripheral cells and neural tissue^39^, suggesting that DNA_M_ changes in response to environmental signals appear to be system-wide. Third, in accordance with previous studies, the heterogeneous mixture of cell types in whole blood samples used for *FKBP5* DNA_M_ analyses may constitute a potential confound.

In conclusion, the current study provides no evidence for an association of *FKBP5* intron 7 DNA_M_ status with CT and cortisol output, at least not in a healthy, generally more resilient sample. Future studies on traumatized/unexposed individuals both with and without stress-related disorder might help to further disentangle effects of CT on *FKBP5* DNA_M_ and associated HPA-axis dysfunctions from those related to psychiatric conditions.

## Supplementary information

Supplementary Table 1

Supplementary Table 2

Supplementary Table 3

Supplementary Table 4

Supplementary Figure 1
