# Supplementary material for: No association between FKBP5 gene methylation and acute and long-term cortisol output
Source: Transl Psychiatry. 2020 Jun 2;10:175. doi: 10.1038/s41398-020-0846-2 (PMC7266811; doi:10.1038/s41398-020-0846-2)
Supplement: Supplementary file 1 — Supplementary Table 1 [file 41398_2020_846_MOESM1_ESM.doc]

**No association between *FKBP5* gene methylation and acute and long-term cortisol output**

**Supplementary Table 1:** Correlation between DNA methylation in *FKBP5* intron 7 bin 2 and childhood trauma according to the childhood trauma questionnaire (CTQ) in the overall sample.

|  | *FKBP5* CpG site 1 methylation | | *FKBP5* CpG site 2 methylation | | *FKBP5* CpG site 3 methylation | | average *FKBP5* methylation | |
| --- | --- | --- | --- | --- | --- | --- | --- | --- |
|  | r | p | r | p | r | p | r | p |
| emotional abuse | -.084 | .238 | -.172* | .015 | .125 | .080 | -.103 | .148 |
| physical abuse | -.060 | .398 | -.168* | .018 | .136 | .056 | -.088 | .218 |
| sexual abuse | -.118 | .096 | -.138 | .052 | .070 | .329 | -.110 | .121 |
| emotional neglect | -.034 | .663 | -.099 | .165 | .042 | .553 | -.069 | .331 |
| physical neglect | -.122 | .085 | -.120 | .091 | .093 | .189 | -.086 | .227 |
| CTQ sum | -.096 | .178 | -.171* | .016 | .113 | .113 | -.111 | .118 |

Note: *p < 0.05 uncorrected for multiple comparison
