# Supplementary material for: No association between FKBP5 gene methylation and acute and long-term cortisol output
Source: Transl Psychiatry. 2020 Jun 2;10:175. doi: 10.1038/s41398-020-0846-2 (PMC7266811; doi:10.1038/s41398-020-0846-2)
Supplement: Supplementary file 2 — Supplementary Table 2 [file 41398_2020_846_MOESM2_ESM.doc]

**No association between *FKBP5* gene methylation and acute and long-term cortisol output**

**Supplementary Table 2:** Correlation between DNA methylation in *FKBP5* intron 7 bin 2 and childhood trauma according to the childhood trauma questionnaire (CTQ) in *FKBP5* rs1360780 risk (T) allele carriers (N=77).

|  | *FKBP5* CpG site 1 methylation | | *FKBP5* CpG site 2 methylation | | *FKBP5* CpG site 3 methylation | | average *FKBP5* methylation | |  |
| --- | --- | --- | --- | --- | --- | --- | --- | --- | --- |
|  | r | *p* | r | p | r | p | r | *p* | |
| emotional abuse | .042 | .717 | -.048 | .679 | .130 | .259 | .056 | .627 |  |
| physical abuse | -.020 | .861 | -.140 | .224 | .136 | .238 | -.017 | .882 |  |
| sexual abuse | -.339^#^ | .003 | -.070 | .545 | .077 | .506 | -.086 | .457 |  |
| emotional neglect | .011 | .926 | .046 | .689 | .018 | .874 | .044 | .702 |  |
| physical neglect | -.043 | .709 | -.012 | .920 | .068 | .559 | .022 | .852 |  |
| CTQ sum | -.017 | .886 | -.027 | .815 | .099 | .393 | .036 | .753 |  |

^#^p < 0.05, Bonferroni-corrected for three CpG sites x five CTQ dimensions
