# Supplementary material for: No association between FKBP5 gene methylation and acute and long-term cortisol output
Source: Transl Psychiatry. 2020 Jun 2;10:175. doi: 10.1038/s41398-020-0846-2 (PMC7266811; doi:10.1038/s41398-020-0846-2)
Supplement: Supplementary file 3 — Supplementary Table 3 [file 41398_2020_846_MOESM3_ESM.doc]

**No association between *FKBP5* gene methylation and acute and long-term cortisol output**

**Supplementary Table 3:** Correlation between DNA methylation in *FKBP5* intron 7 bin 2 and childhood trauma according to the childhood trauma questionnaire (CTQ) in individuals with the protective (CC) rs1360780 genotype (N=98).

|  | *FKBP5* CpG site 1 methylation | | *FKBP5* CpG site 2 methylation | | *FKBP5* CpG site 3 methylation | | average *FKBP5* methylation | |  |
| --- | --- | --- | --- | --- | --- | --- | --- | --- | --- |
|  | r | *p* | r | p | r | p | r | *p* | |
| emotional abuse | -.106 | .302 | .142 | .165 | .010 | .926 | .068 | .510 |  |
| physical abuse | -.059 | .569 | .032 | .759 | .093 | ,367 | .049 | .631 |  |
| sexual abuse | -.024 | .812 | .079 | .443 | .030 | .774 | .062 | .546 |  |
| emotional neglect | .012 | .906 | .080 | .435 | -.026 | .802 | .047 | .646 |  |
| physical neglect | -.063 | .541 | .052 | .613 | .001 | .992 | .015 | .883 |  |
| CTQ sum | -.059 | .565 | .112 | .273 | .010 | .926 | .063 | .539 |  |
