# Supplementary material for: No association between FKBP5 gene methylation and acute and long-term cortisol output
Source: Transl Psychiatry. 2020 Jun 2;10:175. doi: 10.1038/s41398-020-0846-2 (PMC7266811; doi:10.1038/s41398-020-0846-2)
Supplement: Supplementary file 4 — Supplementary Table 4 [file 41398_2020_846_MOESM4_ESM.doc]

**No association between *FKBP5* gene methylation and acute and long-term cortisol output**

**Supplementary Table 4:** Correlation between childhood trauma according to the childhood trauma questionnaire (CTQ) and cortisol stress reactivity to the Trier Social Stress Test (AUCg) and hair cortisol concentrations (HCC).

|  | cortisol AUC_g_^1^ | | HCC^2^ | |
| --- | --- | --- | --- | --- |
|  | r | *p* | r | *p* |
| emotional abuse | -.016 | .818 | -.062 | .390 |
| physical abuse | .036 | .612 | -.009 | .902 |
| sexual abuse | -.141 | .048* | .021 | .769 |
| emotional neglect | .006 | .936 | -.048 | .509 |
| physical neglect | -.078 | .791 | .062 | .397 |
| CTQ sum | -.034 | .639 | -.025 | .728 |

Notes: ^1^Analyses were corrected for oral contraceptive use, smoking status and baseline cortisol levels; ^2^analyses were corrected for age, sex, oral contraceptive use, hair treatment, hair washing frequency; *p < 0.05 uncorrected for multiple comparison.
