# Supplementary material for: No association between FKBP5 gene methylation and acute and long-term cortisol output
Source: Transl Psychiatry. 2020 Jun 2;10:175. doi: 10.1038/s41398-020-0846-2 (PMC7266811; doi:10.1038/s41398-020-0846-2)
Supplement: Supplementary file 5 — Supplementary Figure 1 [file 41398_2020_846_MOESM5_ESM.doc]

**No association between *FKBP5* gene methylation and acute and long-term cortisol output**

**Supplementary Figure 1:**


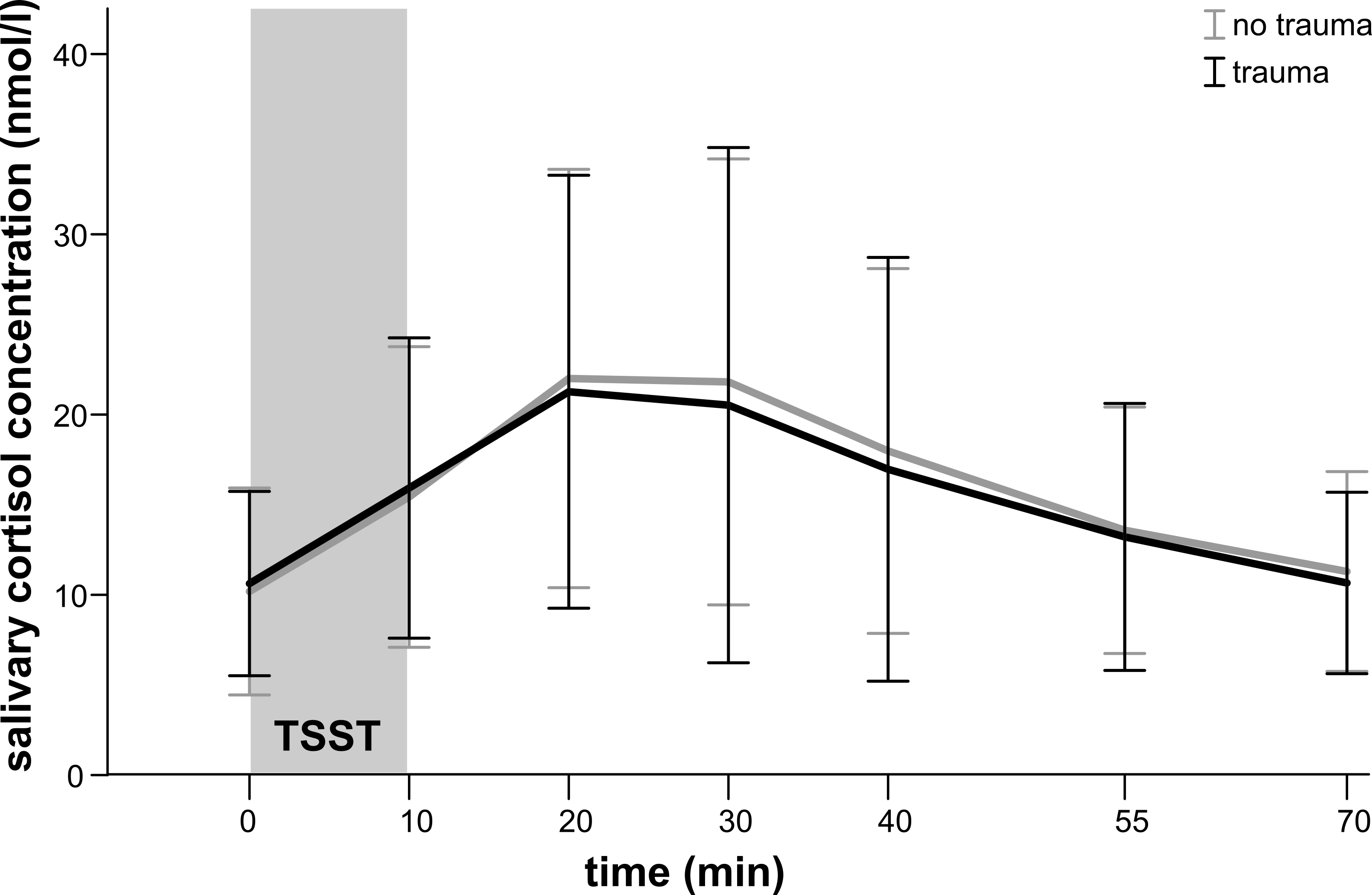


Figure S1: Mean salivary cortisol concentrations in response to the Trier Social Stress Test (TSST) as a function of moderate-severe childhood trauma. Values represent Mean ± 1 SD. Please note that this figure shows raw values for illustrative purposes; the statistical analyses were performed on log-transformed cortisol concentrations.
